# ‘I Don't Think They've Ever Seen People Like Me Do Jobs Like This’: Exploring Hope Within Strengths‐Based Employment Services for People With Disability

**DOI:** 10.1111/jar.70147

**Published:** 2025-11-02

**Authors:** Kelly Carr‐Kirby, Sean Horton, Chad A. Sutherland, Victoria Paraschak, Patricia Weir

**Affiliations:** ^1^ Department of Kinesiology University of Windsor Windsor Ontario Canada

**Keywords:** employment services, hope, inclusion, intellectual disabilities, social change, strengths perspective

## Abstract

**Background:**

Strengths‐based employment services focused on the abilities of people with intellectual disabilities challenge traditional, deficits‐based orientations. Within the presence of hope, which sustains collective effort toward a preferred future, such employment services may stimulate social change. Therefore, the presence of hope was examined within strengths‐based employment settings for adults with intellectual disabilities to understand its potential to establish inclusive workplaces.

**Methods:**

Employees with intellectual disabilities supported by strength‐based employment services, as well as their employers and co‐workers, completed semi‐structured interviews. Data were analysed using thematic analysis.

**Results:**

Hope was present within strength‐based employment services through the (a) co‐sharing of strengths, (b) movement toward a shared, preferred future, (c) alignment of personal and collective goals, and (d) (co)transformation of interacting parties.

**Conclusion:**

Implementing strengths‐based employment services for people with intellectual disabilities is supported, as it facilitates the cultivation of hope and thus movement toward inclusive and equitable workplaces.

**Summary**

- People with intellectual disabilities can use their strengths to complete important tasks in the workplace.
- Hiring employees with intellectual disabilities for their ability to perform workplace tasks counters deficits‐based approaches to disability.
- Using strengths‐based hiring practices encourages inclusive and equitable workplaces where the abilities of people with disability are valued.
- Movement toward inclusive workplaces is possible through the presence of hope, which creates a collective effort to attain a preferred future.

## 1 Introduction

Developmental service sector agencies have traditionally assisted people with disabilities gain employment through a social service model, where employers hire for charitable reasons, as a corporate social responsibility, or as a strategy to improve the company's public image (Dale 2004; Dwertmann et al. 2021; Institute for Corporate Productivity [ICP] 2014; Lysaght et al. 2012). Reflecting a deficits‐based approach, where pathology, impairment, and abnormality are emphasised to prompt action (Bartlett 1958; Saleebey 2009; Weick et al. 1989), this method (re)produces low expectations and limited employment profiles of people with intellectual disabilities (Berrigan et al. 2020; Crawford 2011; Winsor et al. 2018). These assumptions and stereotypes reflect perceptions of dependence, minimal capacity to develop skills, and an inability to perform workplace responsibilities, which create a significant barrier to employment (Bonaccio et al. 2020; Ju et al. 2013; Vornholt et al. 2018).

When employment services subscribe to a traditional approach rooted in a deficits‐based perspective, and employer practices exemplify charitable attitudes nuanced by (un)intentional discrimination, the value of employees with intellectual disabilities is reduced. Minimising their potential contribution ignores evidence that suggests people with intellectual disabilities are an untapped talent pool of motivated and dependable employees, with lower levels of absenteeism, higher rates of productivity and retention, and the ability to stimulate customer loyalty and workplace morale (Institute for Corporate Productivity [ICP] 2014; Fisher and Connelly 2020; Lindsay et al. 2018; Lysaght et al. 2012). High‐performance organisations, ranking in the top quartile of Market Performance Index scores, have acknowledged this assertion, as they pursue employees with intellectual disabilities for business benefits, recognising that a worker with intellectual disabilities can be dependable, engaged, and motivated, while offering exemplary attendance, attention to work quality, and high productivity (Institute for Corporate Productivity [ICP] 2014).

Implementing a strengths‐based perspective (Saleebey 2009) challenges the traditional, deficits‐based framework used by employment service agencies. Aligning with the recommendation of the World Health Organisation, potential employers should be addressed on a business level, rather than a social service level, to improve employment outcomes for people with disabilities by altering discriminatory attitudes within the workplace (World Health Organization 2011). In doing so, the abilities and skills of job seekers with intellectual disabilities are presented to employers as a desirable job match to satisfy workplace requirements and are therefore viewed as active and valued contributors to the labour force. Employers are prompted to hire for business‐wise decisions, which elicits a recognition that all people, regardless of (dis)ability, have strengths. Such employment services demonstrate an exploration, identification, and utilisation of an individual's abilities when entering the labour market (Bartlett 1958; Saleebey 2009; Weick et al. 1989). Framing the situation to focus on potential (Anderson and Heyne 2013; Paraschak and Thompson 2014) counters a medical model of disability that emphasises pathology and impairment (Bartlett 1958; Saleebey 2009; Weick et al. 1989) and creates space for greater understanding, empowerment, and social change (Paraschak 2013a; Paraschak and Thompson 2014). When grounding the revitalization of employment services within a strengths perspective, people with intellectual disabilities are presented as valuable employees based on their existing strengths, as well as their unlimited capacity to gain and enhance skills and competencies (Saleebey 2009). While traditional methods, often encumbered by discriminatory, exclusionary, or stereotypical practices, may underestimate the abilities and potential for growth of employees with intellectual disabilities, strengths‐based employment services realise individual abilities prevail (Saleebey 2009) with such challenges providing opportunity to flourish (Masten 2001; Saleebey 2009). Throughout the process, support staff and employers work *with* the job seeker to collaboratively identify and concurrently benefit from the individual's strengths while utilising the resources within the given workplace for further development of those strengths (Saleebey 2009).

Such strengths‐based employment for individuals with disability remains novel, with minimal research yet to assess its application to improve employment outcomes (e.g., Johnson 2022). While positive psychology and strengths‐based approaches have been introduced more broadly to the field of disability (i.e., Wehmeyer 2013; Niemiec et al. 2017), it remains early in its inception as social services, in particular social work practices, have traditionally been preoccupied with the identification and ‘fixing’ of problems (Ur and Ganie 2022; Weick et al. 1989). However, in the 1950s, the importance of recognising strengths of individuals and communities was identified. A rise in the application of a strengths‐based approach followed, as it aligned with the values within the practice of social work, such as self‐determination, empowerment, and recognising an individual's inherent worth and dignity (Ur and Ganie 2022; Weick et al. 1989).

Processes related to this perspective can be further operationalised through the integration of practices of hope, as the identification and enhancement of individuals' strengths can be supported through its cultivation (Jacobs 2005; Paraschak 2013b; Upham 2019). Relational hope, or a ‘hope‐in’ orientation, exists as a joining together of people in collective reflection and action to attain a shared, preferred future (Jacobs 2005), where the co‐transformation of all interacting parties is possible (Jacobs 2005, 2008; Paraschak and Heine 2019). Hope, or the communal effort to reach a preferred future, is represented within strengths‐based employment services as job seekers with intellectual disabilities, as well as their employers, co‐workers, and the developmental service sector agencies collectively move toward inclusive and equitable workplaces. Further connecting practices of hope to a strengths perspective, particularly its emphasis on resources, is the co‐creation of a hope‐enhancing environment to aid movement toward communal aspirations. Within this context, all individuals serve as human resources to one another by proactively sharing their strengths while drawing on the strengths of others as they seek to attain a collective goal (Maxwell et al. 2022; Paraschak 2013b; Paraschak and Kossuth 2021; Upham 2019). In this instance, a person with a disability transitions from being labelled as ‘helped’ to ‘helper’, thus creating a relationship of equality where we recognise the common humanity among all people (Jacobs 2008; Saleebey 2009). While this environment exemplifies a relational, ‘hope‐in’ orientation (Jacobs 2005, 2008), a contrasting, yet complementary ‘hope‐for’ approach (Jacobs 2005, 2008; Snyder 2002) can contribute to the attainment of the imagined future. This expression of hope focuses on the attainment of personal goals and desires as an individual's achievement dominates visions for the future (e.g., attaining a promotion; Jacobs 2005). Despite the individualistic nature of a ‘hope‐for’ orientation, movement toward the shared future can be further supported when individual and collective goals align. Such alignment can lead to a greater co‐sharing of individual strengths as all parties realise their contributory efforts will support the concurrent achievement of personal and communal goals (Anderson 2015; Upham 2019). Therefore, when an individual's personal desires can be achieved through the attainment of the preferred future, the extent of their engagement in the communal effort to reach the shared, preferred future may increase (Upham 2019).

Given that practices of hope intend to progress toward a preferred future, this perspective assumes an ever‐changing world where human agency can influence and transform current circumstances (Freire 2002; Jacobs 2005, 2008). Aligning with a social constructionist epistemology, we recognise that meaningful reality is generated by human practices (Crotty 1998) and disability is constructed through interactions between humans and their world (Oliver 1992). Restrictive social structures create parameters to imaginable possibilities envisioned for individuals with disabilities, which, for example, are illustrated by their limited employment profiles (Giddens 1984; Metcalfe 1993; Ponic 2000). With minimal conscious consideration, behaviours and assumptions aligned with exclusionary societal boundaries are continuously reproduced (Giddens 1984; Paraschak 2000), including ‘charitable’ hiring practices that dismiss existing strengths and potential contributions to the workplace by employees with intellectual disabilities.

Alteration of disabling societal boundaries is possible through acts of agency that challenge prevailing social structures and undermine naturalised behaviours and assumptions (Giddens 1984; Paraschak 2000). For example, negative attitudes toward disability may be amended through greater exposure to people with intellectual disabilities, specifically when actions of these individuals challenge imaginable possibilities given the existing social boundaries (Tregaskis 2003, 2004). Such behaviours can alter one's practical consciousness, or implicit understanding of how to act within the social world, as naturalised assumptions associated with disability are opposed (Giddens 1984). Therefore, when strengths‐based employment services for job seekers with intellectual disabilities counter the traditional deficits‐based social structures that minimise workforce participation of people with disability (Berrigan et al. 2020; Butterworth et al. 2015; Crawford 2011; Dale 2004; Fesko et al. 2012; Institute for Corporate Productivity [ICP] 2014; Lysaght et al. 2006; Lysaght et al. 2012; Vornholt et al. 2018; Zwicker et al. 2017), social change may occur via the transformation of one's practical consciousness as we recognise an alternative possibility for employees with intellectual disabilities (Giddens 1984). During such interactions, Jacobs (2008) contends we must “put aside what we *think* we know” to prepare ourselves for co‐transformation (emphasis added, p. 569).

Through this process, our objective is to reduce exclusionary, discriminatory, and stereotypical attitudes toward job seekers and employees with intellectual disabilities by shifting from a deficits‐based model of disability to a strengths‐based perspective (Anderson and Heyne 2013; Giddens 1984; Saleebey 2009). Ensuring employees with intellectual disabilities are hired for their personal skillset, strengths‐based employment services dismiss charitable hiring practices of people with disability. Rather, applying a Strengths and Hope perspective facilitates the identification and utilisation of strengths, for which employees with intellectual disabilities are hired. This opposes the prevailing practical consciousness grounded in a deficits‐based approach and facilitates social change through collective movement toward the preferred future of inclusive and equitable workplaces (Paraschak 2013b). Emphasising strengths allows dominant, deficits‐based assumptions of disability to be challenged, while hope sustains collective effort toward a communal goal. While strengths‐based employment services incorporate a strengths perspective (Saleebey 2009), social change also requires the existence of hope; that is, the joining together of people in collective reflection and action to attain a shared, preferred future. Therefore, the objective of the study was to examine the presence of hope within strengths‐based employment settings for adults with intellectual disabilities to understand its potential to stimulate social change related to inclusive workplaces.

## 2 Methods

### 2.1 Participants

Following clearance from the host university's Research Ethics Board, purposeful sampling (Patton 2002) was used by two local developmental service sector agencies to recruit employees with intellectual disabilities and employers involved with their respective strengths‐based employment services. The employment services assured the use of a strengths‐based approach as the social service agencies were fulfilling a provincially sponsored grant to revitalize their employment supports to assist individuals with intellectual disabilities in gaining meaningful employment based on their strengths that could fulfill specific workplace roles. Therefore, only employees and employers associated with this provincially funded project were recruited as study participants. Inclusion criteria included: (1) participation in strengths‐based employment services supported by the provincially funded grant to revitalize current employment supports to hire people with intellectual disabilities for their skills to fulfill workplace roles, (2) participation with these employment services for eight weeks or longer, (3) participants satisfy the age requirement of 18 years of age or older, and (4) the requirement of employees to be diagnosed with an intellectual disability, while employers and co‐workers not be diagnosed with an intellectual disability. The social service agency connected with local businesses to educate and support their involvement with strength‐based hiring practices for people supported by their employment service program. Recruitment for this study involved email and in‐person contact with all local business employers and employees supported by these strength‐based employment services. Co‐workers of employees with intellectual disabilities were then recruited through snowball sampling that extended from participating employers (Patton 2002). The initial connection included the provision and explanation of the Letter of Information (either electronically or hard copy), which outlined the purpose, procedures, and voluntary nature of the study, its contribution to the doctoral dissertation of the first author, as well as benefits, risks, compensation, confidentiality, and withdrawal of participation. Once potential study participants agreed to be contacted by the research team, the social service agency provided contact information to the first author. Study participants included 16 employees with intellectual disabilities (10 males, 6 females; M_age_ = 31.7 years; age range = 19 to 58 years), 12 employers who participated in hiring processes and regularly oversaw employee performance (6 males, 6 females; M_age_ = 50.8 years; age range = 40 to 59 years), and 12 co‐workers (6 males, 6 females; M_age_ = 40.3 years; age range = 19 to 62 years) of employees with intellectual disabilities. More interviews (16 interviews compared to 12 interviews) were conducted with employees with intellectual disabilities compared to other participants, since interviews with employees with intellectual disabilities were shorter and therefore yielded less data. The decision to add more interviews was made during the data collection process to assist with meeting data saturation during analysis. While some employees, employers, and co‐workers came from the same workplace, this was not a requirement for study participation. Among the 40 participants, 4 participants with intellectual disabilities were previously acquainted with the research team, as they participated in a community program in which the researchers were involved. All other participants were previously unknown to the research team. To see additional participant information, including educational attainment, employment sector, employment position, duration of employment, and weekly employment hours, please see Table 1.

**TABLE 1:** Participant information.

Participant group	Highest level of education	Place of employment	Employment position	Duration of employment (average in years)	Employment schedule (average hours per week)
Employee	Elementary School (*n* = 1) High school (*n* = 13) College (*n* = 2)	Municipality (*n* = 4) Sales and services (*n* = 9) Health and medical (*n* = 2) Social services (*n* = 1)	Janitorial (*n* = 5) Food preparation (*n* = 5) Assistant (*n* = 1) General labourer (*n* = 2) Crossing guard (*n* = 1) Not answered (*n* = 2)	2.1	15.8
Employer	College (*n* = 5) University (*n* = 7)	Municipality (*n* = 4) Sales and services (*n* = 5) Heath and medical (*n* = 2) Social services (*n* = 1)	Manager (*n* = 6) Supervisor (*n* = 1) Owner (*n* = 4) Chief (*n* = 1)	10.7	49
Co‐worker	High school (*n* = 5) College (*n* = 5) University (*n* = 2)	Municipality (*n* = 2) Sales and services (*n* = 5) Health and medical (*n* = 4) Social services (*n* = 1)	Janitorial (*n* = 2) Food preparation (*n* = 2) Assistant (*n* = 3) General labourer (*n* = 3) Professional title (*n* = 1) Union representative (*n* = 1)	10.8	37.6

### 2.2 Procedures

Prior to the commencement of each interview, the first author reviewed the consent form with the participant, ensured written consent was obtained, followed by verbal consent by the participant on the audio recording. Each participant then completed a one‐on‐one semi‐structured interview framed within a Strengths and Hope perspective (Anderson 2015; Paraschak 2013b). Anderson (2015) provided the interview guide, which was previously used to explore the experience of participants with disability involved in an adapted sailing program through a Strengths and Hope perspective. While questions were revised to focus on the employment experience, the essence of questions remained the same as Anderson's (2015) original interview guide. Main topics of the interview guide included (1) background and general questions (e.g., “Can you tell me about your experience working at [insert business name]?”), (2) questions from a strengths perspective (e.g., “What strengths did you have starting your job?”), (3) questions based on practices of hope (e.g., “How has your job changed your outlook for the future?”), and (4) questions based on a social model of disability (e.g., “How has being employed reinforced and/or changed your perceptions about disability?”). While the interview questions remained the same for participants within different groups (i.e., employees, employers, co‐workers), phrasing was altered to ensure questions reflected each person's role within the workplace.

All interviews were conducted by the first author (female, PhD Candidate, with six years of interviewing experience) who maintained field notes throughout interviews. Open‐ended interview questions and non‐directive probes allowed participants to elaborate on responses through incorporation of details and illustrative stories, while allowing the freedom to explore new, emerging topics (Patton 2002). Interviews were completed in a private room at the host university (*n* = 2), participating developmental service sector agencies (*n* = 8), participant workplaces (*n* = 24) or homes (*n* = 5), as well as within a fast‐food chain restaurant (*n* = 1). The interview that was completed at a fast‐food restaurant was conducted at this specific location due to a direct request from the participant. Interview duration ranged from 8 min 34 s to 62 min 7 s (M = 33 min, 32 s), and yielded 22 h 14 min of audio recording.

### 2.3 Data Analysis

Collected data were examined by the first author using thematic analysis (Braun and Clarke 2006; Braun et al. 2016). Typed transcripts were reviewed and corrected against audio recordings to ensure verbatim accuracy. Initial codes were assigned to recurrent concepts that appeared across transcripts and were meaningfully related to the research question. Initial codes were merged and organized to develop a detailed, theoretically grounded thematic map that outlined emergent themes and sub‐themes. Internal homogeneity and external heterogeneity were assessed, ensuring each data extract met criteria to be within a single theme, while being explicitly unfit to be categorised among others. Defining each theme prompted the recognition of its ‘essence’ or ‘story’ as valuable to the overall findings (Braun and Clarke 2006). All authors had an opportunity to review the organization of codes, as well as examples of data extracts (i.e., participant quotes) to ensure agreeance with coding decisions. Feedback received from co‐authors prompted the iterative process of revising initial codes, re‐organization of data extracts within themes, and further defining the themes in a way that meaningfully addressed the research question. Provided feedback yielded continuous iterations of this process until all authors reached consensus. While most revisions to the data analysis were completed through the iterative process of review and revise, details, such as the appropriateness of data extracts within each theme, as well as the ability of the data to address the research question, continued to be adjusted throughout manuscript preparation. Data analysis procedures, as well as data saturation, were completed and met, respectively, for each participant group. Results for each participant group were then combined at the level of interpretation during the write‐up of the results section, with each theme being independently present among each participant group. Criteria provided by Tracy (2010) were used to assess methodological quality.

## 3 Results

Participants' perspectives, descriptions, and lived experiences related to strengths‐based employment settings for adults with intellectual disabilities evidenced the existence of hope, along with the potential to stimulate social change through the development of inclusive workplaces. Practices of hope identified within the dataset have been organized into four themes:

1. *Co‐sharing strengths to be valuable human resources for one another*, where individuals used their abilities to contribute to the attainment of collective goals.
2. *Movement toward a shared, preferred future of inclusive workplaces* was showcased as a communal vision that offered meaningful, competitive employment positions for people with intellectual disabilities while fulfilling important workplace roles.
3. *Aligning collective and personal goals to achieve inclusive workplaces*, where all participant groups identified personal goals related to their involvement with employment services for people with intellectual disabilities, which contributed to the attainment of the preferred, collective future.
4. *Possibility for the co‐transformation of assumptions related to disability*, which was possible through participant groups being open during interactions with one another, leading to the alteration of one's practical consciousness surrounding disability.

### 3.1 Co‐Sharing Strengths to Be Valuable Human Resources for One Another

Strengths of employees with intellectual disabilities acted as human resources to others, as they used their abilities to complete specific and necessary tasks within the workplace, as identified by employer, LOKA, “she was able to take on tasks that we had as part of daily operations and become a valuable member of the workforce… she's doing things that we would hire someone to do”. Similarly, co‐workers identified how employees with intellectual disabilities acted as human resources by sharing work responsibilities. Illustrating this situation within the health field, co‐worker AMRC explained:

She's made my life a little easier. What she does for me is, like, there are some meds in boxes that need to be popped out. Before I'd have to pop them out when the machine needed it, and that was very time consuming, so I have her do it… so [she's] honestly made my life a whole lot easier.

Employees with intellectual disabilities identified the reciprocal relationship necessary for the co‐construction of a hope‐enhancing environment when they drew on the strengths of others to reach the preferred future of an inclusive workplace. Primarily, employees with intellectual disabilities depended on employers to provide training and information on how to properly complete job responsibilities, as described by employee, RYRI, “my boss… because she told me what to do, and how to do it. She's showed me some mistakes I did and helped me fix them”, and employee, FIHA:

It's mostly my boss that kind of helps me in the end, you know, like she shows me how to do this the right way instead of the wrong way, so, for next time, I know how to do it that way.

Beyond instruction of technical skills, employees with intellectual disabilities relied on employers' abilities to be a resource for support, as explained by employee, ERDI, who recognised her employer as her main human resource, “at my job it would be my boss, like if you have any questions you can ask, just feeling that there is someone there to talk to, like you're not by yourself”, and employee, MIFA, “if I'm having a hard time at work, [my boss] will take the time to re‐iterate what I have to do, and he's patient with me… I've never felt more comfortable than in this job”.

Co‐sharing of strengths was also evidenced within the relationship of employees with intellectual disabilities and their co‐workers. While co‐workers benefited from the abilities of employees with intellectual disabilities, co‐workers also offered workplace‐related strengths that assisted employees with intellectual disabilities when completing specific aspects of their job, as explained by employee, JUCH, who worked in the food service industry:

[Co‐worker] is one of the ladies that does prep, [co‐worker] is another prep person, and [co‐worker] taught me, he was basically my trainer on how to do dishes at work. They've helped me learn how to portion out stuff and use different bags.

Employees with intellectual disabilities further drew on their co‐workers to serve as a supportive resource that provided comfort and friendship, as expressed by an employee with intellectual disabilities, EVPE, “the confidence [I gained] I think is from the people that I work with, like they made me feel comfortable.”

### 3.2 Movement Toward a Shared, Preferred Future of Inclusive Workplaces

All participant groups identified a shared vision of inclusive workplaces, which was supported by an understanding of the common humanity of all people, witnessing employees with intellectual disabilities exceed expectations, and an acknowledgement of a workplace without barriers to equal participation. A communal vision for the future, which aligned with the intentions of the associated employment services, was evident among employees with intellectual disabilities, employers, and co‐workers. With the mutual goal emphasising inclusive workplaces offering meaningful employment for people with intellectual disabilities, contributory efforts from employers were recognised as a desire to hire more employees with intellectual disabilities, as noted by employer, SAPE, “I'm open to hiring [people with intellectual disabilities]… if there's something that we can teach them, teach anybody with a disability to do in here, I'm all for it.” Similar views were shared among co‐workers, who embraced an opportunity to work alongside more individuals with intellectual disabilities, such as co‐worker, KECA, who stated, “I think for people with disabilities to come into the workplace, I think it needs to happen more often. I would love to have another person [with intellectual disabilities] come in.” Co‐worker, CAPO, supported this notion when saying, “I just hope that more people with disabilities do find better jobs… I hope more get out in the workforce… I wouldn't mind at all working side by side.” Employees with intellectual disabilities contributed to this vision of the preferred future by explaining a desire for continued employment where they can advance in the workforce, as explained by employee, FIFA:

There are possibilities out there for me. I don't want to do this forever, I just want to keep it for now… I'm still looking for something I'd like to do. There are options out there for me, and I'll find it… lots of possibilities out there.

Building on the desire to advance in the workplace, employee, EVPE, noted her interest in further education to assist in attaining the imagined future:

[Working] made me want to go back to school. I might go back to school and I'd like to do something with pharmacy, learn more about pharmacy so that I can maybe reach something else about medicine.

Employers, co‐workers, and employees with intellectual disabilities recognised the common humanity across people by accepting all members of the community with equal respect. Through the application of these practices of a Strengths and Hope perspective, movement toward inclusive workplaces was possible, as employees with intellectual disabilities were viewed as no different than any other employee, as explained by employer, SUKE:

Why not give [person with intellectual disabilities] a chance, they're the same as everybody. They deserve a chance… you can't treat them any different than anybody else, or else they do feel different themselves. She's no different than anybody else that works here, she's an employee, and she does the same things everybody else does.

Co‐workers further emphasised the commonality among all employees, including those with intellectual disabilities, as explained by co‐worker, SPWI:

[Employee with intellectual disabilities] fits in like any other person here…because she's still an employee, she has the same expectation as any other employee would have. And overall, she's just the same as any other employee. She can function at the same level that we do, at the same pace that we do, with the same quality that we do.

Equality among employees was also recognised by individuals with intellectual disabilities, as they agreed that their treatment was unaffected by their disability, as discussed by employee, LACO, “I thought people would look at me different because of my disability, but I was wrong, they look at me for who I am, not what I have.”

Furthermore, all participant groups identified that employees with intellectual disabilities exceeded expectations, thus forecasting a future where the ability of people with intellectual disabilities is framed in their possibility and potential to be contributing, meaningfully included members of the workforce. Employers identified employees with intellectual disabilities surpassing presumptions about workplace abilities when employer, MAMA, explained:

I think we were initially concerned about the level of work that he'd be doing, or the quality of work that he'd be doing, but I think that's all been thrown out the door, I think he's demonstrated he can do a great job.

Co‐workers who worked closely with a person with intellectual disabilities substantiated this view, as exemplified by co‐worker, RYCO:

People are very quick to start the laundry list of what [an employee with intellectual disabilities] shouldn't do, can't do, hasn't been able to do in the past, and he's exceeded in all areas. He's shown them to be completely ignorant to what he's actually capable of across the board, you know, his abilities… So, I think, don't underestimate somebody, don't assume anything.

Such an enlightening experience noted by employers and co‐workers was mirrored by employees with intellectual disabilities who, following employment, perceived their abilities as greater than they previously imagined, a sentiment they projected on to all people with a disability, as explained by employee, MIFA, “I feel like [employment] is teaching me that somebody like me, or somebody like a person that's disabled, can do anything that they set their mind to”.

Lastly, the removal of disability within a workplace, as per a social constructionist definition (i.e., disability is constructed within the environment), is a testimony to movement toward the preferred future of inclusive workplaces. Specifically, all participant groups indicated that employees with intellectual disabilities could function fully within their job positions, and thus were not disabled by the workplace environment, as described by employer, LOKA, “her success 100% indicates in the right environment [employee with intellectual disabilities] really has no disabilities. We match her skills with tasks… she is 100% doing the job of anybody else… when she's here she has no disability.”

Co‐workers shared similar opinions related to appropriate employee‐workplace fit, as co‐worker, SPWI explained, “it's not really an environment where her impairment has any problems as far as disabling her. As much as we can, we actually enable her to function very easily in our [workplace] environment”. Consistent with this assessment of the workplace, employees with intellectual disabilities agreed with their capacity to function without disability within their places of employment, which was recounted by employee, EVPE:

There are no obstacles because there's not a lot of things that stop me from doing my job. For example, if something was wrong about the environment, they would try to make it so everyone could use it, [work] around it.

### 3.3 Aligning Collective and Personal Goals to Achieve Inclusive Workplaces

While all participant groups identified personal goals related to their involvement with employment services for people with intellectual disabilities, the attainment of each contributed to the preferred, collective future. For example, employers' goals often related to filling an open job position with a reliable employee, regardless of (dis)ability. Since this objective was met through engagement with the employment service agency, it provided meaningful employment for a person with intellectual disabilities and thus was conducive to the preferred, collective future. Employer, DOBE, exemplified this view by indicating her personal goal was simply hiring a loyal employee:

Just finding a loyal staff… just having that person that's going to show up to work every day, and that's what [employee with intellectual disabilities] does, never missed a day unless he was sick. So that's what we were looking for, someone that would be that reliable person, that we could give them a job and they would be there all the time.

Differing in forethought, yet equally instrumental to the shared vision of the future, were employers whose personal goals exhibited intentionality when hiring an individual with a disability, as explained by employer, LOKA:

Initially, you could say it was a very simple goal, having someone with a physical or intellectual disability integrate and sustainably function in our workplace without it seeming like a charity… having someone [with a disability] fit in and sustain their position and be valuable in the workplace.

By emphasising fair and respectful treatment of all employees, co‐workers' personal goals also aligned with the vision for the preferred, collective future. For example, co‐worker, SHEG, stated, “my goal is to make sure that [employees with intellectual disabilities] are treated with dignity and respect, like any other co‐worker would want…it doesn't change”. Similarly, co‐workers sought to ensure a comfortable and desirable work environment for employees with intellectual disabilities, as explained by co‐worker, SPWI:

Basically, [my goal was] just getting [employees with intellectual disabilities] comfortable, making sure that they ask any questions that they need to, and if ever they are uncomfortable, they talk to someone about it. And just making sure that her employment here is a positive experience for her.

Lastly, employees with intellectual disabilities consistently noted goals related to their continued employment and a fair wage, both of which are essential to the prospect of a meaningful and inclusive experience. Expressing the importance of attaining and maintaining employment, employee, AMRE, indicated:

It doesn't give me a purpose in life, if I don't have a job. People become slouches, basically like a couch potato, and you don't want that… [Employment] gives me a purpose in life, and it gives me something to do.

Viewing employment as an avenue for a purposeful life was further shared by employee, FIHA, who also acknowledged a desire for financial independence and the specific opportunities that he aimed to afford:

I didn't want to be bored, you know, I like to be busy, I don't like just sitting around… I need money for a house someday, a car, vacation somewhere, you know. I like to be independent.

### 3.4 Possibility for the co‐Transformation of Assumptions Related to Disability

Co‐transformation between employees with intellectual disabilities and their employers or co‐workers was often manifested through a change in participants' practical consciousness, particularly related to their naturalised assumptions related to the abilities of individuals with disabilities. For example, employees with intellectual disabilities identified an enhanced recognition and confidence in their abilities, which was attributed to their interactions with co‐workers and employers, as explained by employee, AMRE:

They're helping me become more confident in my environment… They've helped me become more confident in who I am. Like, they've taught me a lot…so I'm confident… ‘cause usually I used to be nervous… but now I'm more confident.

Such transformation of employees with intellectual disabilities was substantiated by employers, who noted their role in shaping employees with intellectual disabilities to better recognise their personal abilities, as expressed by employer, DOPA:

Unfortunately [employee with intellectual disabilities] thinks she does a bad job all the time, not that she does a bad job all the time, but she thinks she's never enough, I think slowly she's getting there, because her disability isn't a disability. She is great at what she does.

Similarly, co‐workers acknowledged their role in improving the certainty with which employees with intellectual disabilities viewed their workplace abilities, as explained by co‐worker, SPWI:

Making [employee with intellectual disabilities] more confident… when she first started, she didn't joke a whole lot, but more recently she's been a lot more of an active participant in it and she has a great time with it. She's quite confident at her job.

Alteration to one's practical consciousness surrounding the abilities of people with intellectual disabilities also extended to the co‐transformation of employers and co‐workers. When asked about their role in shaping others, employees with intellectual disabilities, such a MIFA, noted the ability of his actions to challenge existing assumptions:

I think I've showed them that a person that is disabled…like my skills have opened up a whole new world. I don't think they've ever seen people like me do jobs like this. I just feel like I'm opening doors left and right.

Employers also recognised an alteration to their well‐established assumptions related to the abilities of people with disability, as explained by employer, BMRO:

I used to think [disability] was a negative, but this whole thing with [employee with intellectual disabilities] has made me see the advantages, not the disadvantages, that's quite a change for me… always thought of disability as, you know, the ‘dis’ part of disability, instead of the ‘ability’ part.

Comparable to employers' experiences working with a person with intellectual disabilities, co‐workers, too, noted an adjustment to their implicit knowledge about disability, such as co‐worker, AMRC:

Now that I've worked with someone [with intellectual disabilities], I see the potential in everybody like that, like she's capable of a lot… It's very possible that people with intellectual disability, it's harder for them to get jobs and it's harder for them to get places in the world. But when you see people like [employee with intellectual disabilities], you see that it's definitely possible. People [with intellectual disabilities] have talents and they have potential for sure, and people need to realize that.

## 4 Discussion

Identification and utilisation of strengths as human resources during the collective movement toward the preferred future of inclusive workplaces was shared among employees with intellectual disabilities, as well as their employers and co‐workers. Through the cultivation of these practices of hope, (co)transformation, particularly of one's practical consciousness related to disability, was experienced, which indicates the potential of these processes to stimulate social change. While a strengths perspective was evidenced within employment services for people with intellectual disabilities (Saleebey 2009), so too was the presence of hope, as it prompted movement toward the collectively envisioned future (Jacobs 2005, 2008). Hope, as demonstrated by study participants, was manifested through (a) the co‐sharing of strengths, (b) movement toward a shared, preferred future, (c) alignment of personal and collective goals, and (d) the possibility for (co)transformation of interacting parties.

While hope has previously been recognised as a foundational concept to a strengths perspective (Anderson 2015; Freeman‐Gibb 2016; Krawec 2014; Leahy 2020; Paraschak 2013b; Paraschak 2019; Paraschak and Heine 2019; Saleebey 2009; Upham 2019), the present study attends to the distinct role of resources. Employees with intellectual disabilities shared their work‐related strengths, which were drawn upon by employers and co‐workers to have necessary workplace tasks completed. As such, employees with intellectual disabilities were valued as human resources, which likely derived from discarding a deficits‐based, social service model that obscured the abilities of people with impairment. Satisfying the reciprocal behaviours necessary for the presence of hope, employees with intellectual disabilities also drew on their employers and co‐workers as human resources for instruction and support within the workplace. Identification of the importance of human resources to provide emotional and social support aligns with previous studies related to adaptive recreational activity (Anderson 2015), transgender athletics (Leahy 2020), women's participation in sport (Freeman‐Gibb 2016), Sport for Development and Peace (Paraschak and Heine 2019), and men's elite hockey (Upham 2019).

Constructed by openness and mutuality, hope within these workplaces ensued from employees with intellectual disabilities seeking opportunities for integrated employment within the competitive labour market, which conveys a willingness to use their agency to be exemplars of the preferred future (Jacobs 2005, 2008). Committing to these actions resisted dominant social boundaries, as employees with intellectual disabilities acted despite the potential of experiencing workplace discrimination (Crawford 2011; Hernandez et al. 2000; Holzbauer 2004; Morris et al. 2024; Shier et al. 2009; Turcotte 2014; Vornholt et al. 2013), or traditional disincentives for employment, such as the potential loss of monetary support programs (French and Song 2014; Maestas et al. 2013; Wittenburg et al. 2013). Similarly, potential employers and co‐workers had to be willing to commit to the external cause (Jacobs 2005) of inclusive workplaces, despite reservations created by dominant, stereotypical assumptions (Ju et al. 2013; Morris et al. 2024; Vornholt et al. 2018). These collaborative efforts were demonstrated by employees with intellectual disabilities, as well as their employers and co‐workers, who proactively co‐shared personal strengths, such as performing beyond expectations and creating a welcoming environment to establish an inclusive workplace. Sharing and receiving of individuals' strengths was dependent on adopting a position of openness, which ensured participants were receptive to the ability of all others to act as human resources (Jacobs 2008). Such practices of hope facilitated a shift from the deficits‐based orientation by re‐constructing the situation between those with and without impairment through the development of a relationship of equality (Jacobs 2008), which supports the desire of employees with intellectual disabilities to “be treated as an equal” (Voermans et al. 2020, 244). While the person with disability is traditionally assumed to require help (Roush and Sharby 2011), the presence of hope obscured the distinction between ‘helper’ and ‘helped’ (Saleebey 2009) or ‘guest’ and ‘host’ (Jacobs 2008), which created a foundation for recognising the strengths of others, as it was realised that each person in the relationship possessed skills, talents, and assets that could be offered to others as resources (Jacobs 2008; Saleebey 2009).

Progress toward the envisioned future may have been hastened by the purposeful or coincidental alignment of individual and collective goals, as all participants may have been more inclined to co‐share strengths, as such effort would contribute to individual and communal fulfillment (Anderson 2015; Upham 2019). Specifically, during the joint venture to attain inclusive workplaces, personal goals were respected as employees with intellectual disabilities sought integrated, competitive employment, employers intended to hire reliable individuals to fill open positions, and co‐workers desired a positive workplace environment.

Opportunity for individuals with intellectual disabilities to function as exemplar employees derived from the removal of disability from the workplace environment (Barnes 1991; Oliver 1992; Union of the Physically Impaired Against Segregation [UPIAS] 1976). For example, when reflecting on a social model of disability, all participant groups acknowledged that the employee with an impairment was not disabled within the workplace, as the environment and job description allowed them to function like all other typically developing employees. Such elimination of disabling workplace environments aligns with Saleebey's (2009) concept of *membership*, where all people are acknowledged as equal members of humanity (Saleebey 2009), and ensures those with intellectual disabilities do not experience an inequitable decrease in employment due to physical or social constraints imposed upon them due to their impairment. Furthermore, employees with intellectual disabilities functioning without environmentally imposed disability allowed employer and co‐worker expectations to be exceeded, which highlights the principle of a strengths perspective indicating that the upper limits of development are unknown (Saleebey 2009), while also supporting the need to “put aside what we *think* we know” (Jacobs 2008, 569). Taken together, the removal of disability from the workplace environment supported employees with intellectual disabilities to counter dominant, stereotypical assumptions about the abilities of people with impairment by functioning as equal members of the workforce and exceeding preconceived expectations set by employers and co‐workers. These actions, which existed beyond imaginable possibilities for individuals with impairment, challenged prevailing social structures and fostered the possibility for social change through the transformation of the practical consciousness surrounding disability (Giddens 1984). Employees with intellectual disabilities, as well as their employers and co‐workers, identified a co‐transformation to their implicit understanding and naturalised assumptions related to the abilities of people with impairment, which extends findings related to co‐transformation, as previous research has focused on participation in sport and physical activity (Anderson 2015; Freeman‐Gibb 2016; Krawec 2014). Such circumstances and (co)transformative processes derived from strength‐based employment practices support recent research that identifies the impact of disability‐related assumptions and attitudes, as well as the use of accommodations, on workplace experiences for people with intellectual disabilities (Morris et al. 2024).

Taken together, the strengths of the present study include its timely and relevant focus on the employment of people with disabilities, while being theoretically grounded and using multiple perspectives (employees with intellectual disabilities, employers, and co‐workers) to examine the research question. Attainment of data saturation within each participant group, as well as meeting criteria for high‐quality qualitative research (Tracy 2010), assures that this study provides rigorous findings on a practical topic. By merging theory with abundant practical application, this study supports the future use of a Strengths and Hope perspective, not only in research but within practice as well. This is an important strength of the present study, as the application of a Strengths and Hope perspective would aid in the development of inclusion and equity, along with the attainment of goals in a breadth of communal efforts, including boardrooms, sports teams, workplaces, and family units.

While this study provides a meaningful contribution to the field, it is not without limitations. Despite the apparent application of concepts related to a strengths perspective (Saleebey 2009), along with noticeable movement toward the shared, preferred future of inclusive workplaces, the developmental service sector agencies within the study did not intentionally ground their employment services within a Strengths and Hope perspective. Consistent with work by Anderson (2015), where principles of a strengths perspective were evidenced within an adaptive sailing program despite the absence of a formal strengths‐based therapeutic recreation model, it seems the presence of hope can occur organically within policy and practice related to developmental services. However, future work should consider an examination of programming and services consciously developed within this framework, as it may yield greater movement toward the envisioned future (Paraschak and Heine 2019). Furthermore, while this study gathered information from multiple perspectives, it remains limited, and thus it is worthwhile to inquire about additional key members that may contribute to the presence of hope, such as employment support staff and administrators, as well as family members of employees with intellectual disabilities. Overall, the implementation of strengths‐based employment services is supported, as it provides an avenue for the cultivation of hope, which creates the potential for social change and supports collective movement toward inclusive and equitable workplaces.

## Ethics Statement

Ethical clearance, ensuring study compliance with the Tri‐Council Policy Statement: Ethical Conduct for Research Involving Humans, was obtained from the University of Windsor's Research Ethics Board.

## Conflicts of Interest

The authors declare no conflicts of interest.

## Data Availability

The data that support the findings of this study are available from the corresponding author upon reasonable request.
